# Ginsenoside-Rb1 Inhibition of VEGF Release – Structure and Activity Relations (SAR) Perspective

**Published:** 2014

**Authors:** Diana L. Godoy, Brandi S. Betts-Obregon, Andrew T. Tsin

**Affiliations:** Department of Biology, The University of Texas at San Antonio, USA

**Keywords:** Ginsenoside-Rb1, vascular endothelial growth factor (VEGF), ginsenosides

Ginsenoside-Rb1 is a chemically pure steroid glycoside from the root of Panax ginseng, which has a 3000-year history of medicinal use in the Orient.

Betts et al (2011) reported that Rb1 inhibits vascular endothelial growth factor (VEGF) release by retinal pigment epithelial cells, which is a major source of VEGF in the eye (1). Vascular endothelial growth factor is a proangiogenic cytokine that contributes significantly to pathological retinal and choroidal neovascularization in association with diabetic retinopathy and age-related macular degeneration. It is, therefore, highly relevant to study the action of Rb1 inhibition of VEGF release and to further explore an effective method to enhance such an action of Rb1. One possible approach is the chemical modification of this steroid glycoside to increase its potency.

There have been several reports focused on the structure vs. activity relations (SAR) of ginsenosides and their derivatives. By simple acylation of the panaxadiol, an analog of ginsenoside, its anti-tumor activity was significantly enhanced, compared with the non-acylated parent ((Note: Anti-tumor activity was measured by the decrease in the viability of ovarian cancer cell ES-2, osteosarcoma cell U2-OS, human hepatocellular carcinoma cells HepG2 and human lung tumor cell A549) (2).

Using a different cancer cell line (lung fibroblast), Dong et al. (2011) noted that the removal of the sugar moiety (by hydrolysis) significantly increased its cytotoxic potency, based on MTT assays, to measure viable cell numbers (6). Du et al.(2011) also reported that protopanaxadiol with acetyl substitutions had a significant increase in its antiproliferative effect on three human cell types (colorectal, breast and colon cancer cells) and they furthermore attributed this action to an increase of programmed cell death (apoptosis) (3).

Such SAR reports are not limited to chemical modification of ginsenosides leading to inhibition of cancer cell proliferation only. Wei et al (2009) reported that oxidation of panxadiol leads to triperpene derivatives with a significant increase in the ability to inhibit the HIV-1 protease enzyme, which is required for maturation and infectivity of the HIV virus (4). Finally, Wang et al. (2010) reported that epimeric derivatives from 20(S)-panaxadiol have an enhanced pharmacological action to ameliorate myocardial injury induced by isoproterenol in a rat model (5).

Recently, there has been an increased interest in SAR in medicinal chemistry. These reports provide further evidence for the need of exploring how chemically modified ginsenosides may enhance inhibition of VEGF in ocular and other cell types. Such discoveries will provide new knowledge for novel intervention methods to treat angiogenic diseases such as diabetic retinopathy, AMD and cancer.
